# The role of local authorities in health risk governance in Senegal: A One Health perspective

**DOI:** 10.1016/j.onehlt.2026.101620

**Published:** 2026-10-15

**Authors:** Mouhamadou Wilane, Khadija Sandembou Diop, Gérome Sambou

**Affiliations:** a National Global Health Security Program of Senegal, Senegal; b University of Liège, Faculty of Medicine, Belgium

**Keywords:** One Health governance, Local authorities, Health risk, Decentralization, Deconcentration, Senegal

## Abstract

One Health governance in Senegal has been built mainly at the national level through intersectoral platforms, while its territorial dimension remains underexplored. This study aims to analyze the role of local authorities (municipalities and departments) in health risk governance and the institutional reasons for their position.

We conducted a single-case qualitative institutional analysis with four sector-level units of analysis (human, animal, environmental and plant health), based on legal and regulatory texts and on the authors' field experience as members of the national One Health platform. Data were coded in a sector-by-dimension matrix and interpreted with a multi-level governance framework distinguishing deconcentrated state services from decentralized local authorities.

Local authorities hold explicit mandates in human health and in sanitation and waste management, but none in animal or plant health and only partial ones in environmental health. They nonetheless influence health risk determinants through the management of markets, slaughterhouses, waste, stray animals, pastoral resources and urban planning, roles that One Health arrangements do not formally recognize. Their marginalization does not appear deliberate but stems primarily from institutional design rather than capacity deficits: a deconcentrated, sectoral and vertical state architecture and a decentralized, territorial and horizontal system of local governance coexist without formal articulation.

By shifting the focus from national platforms to the territorial level, this study shows that local authorities already exercise de facto functions relevant to One Health. Integrating them requires formal recognition of their role, dedicated resources and functional coordination with regional One Health committees.

## 1 Introduction

Senegal is a West African country with a population of approximately 17 million and a strong reliance on agro-pastoral activities, which engage nearly 30% of the active population and contribute significantly to the national Gross Domestic Product (GDP) [1], [2]. This socio-economic configuration fosters close interactions between humans, domestic animals, and wildlife, creating a favorable environment for the emergence and transmission of zoonotic diseases. Globally, about 60% of emerging infectious diseases are zoonotic, most of them originating in wildlife [3], which underscores the importance of eco-territorial dynamics for understanding contemporary health risks.

In Senegal, several areas illustrate this differentiated territorialization of risks: the eastern regions lie at the interface with the Niokolo-Koba National Park; the northern wetlands (Djoudj National Bird Sanctuary) host large populations of migratory birds; and the groundnut basin is characterized by intense cohabitation between human and animal activities around water resources [4], [5]. Recent outbreaks of highly pathogenic avian influenza (H5N1), Crimean-Congo hemorrhagic fever, Rift Valley fever and Mpox, confirm this spatial heterogeneity of health vulnerabilities [6].

Recognized as a regional pioneer in the implementation of the One Health approach, Senegal established the National Global Health Security Council (*HCNSS-OH*) in 2017, attached to the Prime Minister's Office [7]. This institutional choice aims to ensure political visibility and legitimacy for the national multisectoral health security program [8] and strengthened multisectoral coordination at the central level, as illustrated during the COVID-19 response [9].

However, the centralization of governance at this level structurally reinforces a top-down model. The evaluation of the National Action Plan for Health Security (NAPHS 2018–2023), through the International Health Regulations (IHR) Joint External Evaluation tool, explicitly identified the operationalization of health security at the local level and the effective implementation of regional plans among the key challenges [10].

Efforts toward territorialization have nevertheless been initiated through regional One Health security committees *(CRSS-OH)*. These platforms are led by regional governors and bring together deconcentrated state services, local authorities, communities, and civil society actors [7]. In parallel, Senegal has pursued a decentralization process since independence, marked by three major reforms (Acts I, II, and III). Act III (2013) granted increased responsibilities to local authorities in the field of human health, but similar prerogatives have not been extended to animal or environmental health, and associated resources remain limited [11], [12].

The One Health governance literature has focused mainly on national intersectoral platforms and international coordination [13], [14], and the territorial level remains underexplored in empirical studies in West Africa. Senegal is an especially informative case because it combines one of the earliest institutionalizations of One Health in the region with one of its most advanced decentralized governance systems [15].

This article examines the role of local authorities in health risk governance in Senegal by analyzing the institutional architecture of the One Health approach alongside the decentralization framework. It contributes to the One Health literature in three ways. First, it examines the territorial dimension of One Health governance in a decentralized African context, shifting the analytical focus from national platforms to subnational level. Second, it shows that the marginalization of local authorities is primarily due to institutional design rather than to capacity deficits, and that these authorities already influence health risk determinants through their mandates in waste management, public hygiene, urban planning, livestock markets and natural resource management. Third, it proposes concrete conditions, and operational entry points, for integrating local authorities into One Health governance mechanisms.

## 2 Conceptual framework

### 2.1 Territorial governance and multi-level One Health dynamics

Territorial governance refers to all the processes by which public and private actors coordinate their actions at the level of a given territory or a defined spatial anchor (municipality, department, region). It is characterized in particular by a mixed proximity (geographical and institutional) and democratic accountability [16], [17]. These features make it a particularly relevant level for addressing complex and cross-sectoral issues such as health risks.

One Health governance has historically developed with a reactive orientation, largely in response to major health crises (Ebola, avian influenza, COVID-19), and according to a sectoral and functional logic centered on coordination between ministries and international organizations [13].

Recent studies confirm that One Health governance arrangements remain largely centralized and reactive, with limited involvement of subnational levels [13], [14], despite the broader and more integrative definition proposed by the One Health High-Level Expert Panel (OHHLEP) [18].

We analyze this tension through the multi-level governance framework of Hooghe and Marks [19], which distinguishes:

- Type I – governance rests on general-purpose, stable, and non-overlapping territorial jurisdictions, which corresponds to the logic of decentralized local authorities;
- Type II – governance relies on flexible, task-specific and functionally defined structures, characterized by their purpose rather than their territorial anchorage, which reflects the dynamics of Senegal's One Health platform.

The two logics coexist without a formal mechanism of articulation, a situation that echoes a broader mismatch in contemporary health governance between the spaces where risks emerge and those where decisions are made [20].

### 2.2 Understanding the difference between deconcentration and decentralization

The distinction between deconcentration and decentralization is central to the argument [21] and is applied consistently throughout the article. We use the following terms strictly:

- Deconcentrated state: governors (who chair the Regional Development Committees, *CRD*) and prefects (who coordinate the Departmental Development Committees, *CDD*) are representatives of the central state; regional and departmental technical services (health, veterinary, environment, plant protection) are deconcentrated sectoral structures. Both are appointed state officials whose legitimacy is hierarchical.
- Decentralized local authorities: municipalities and departments are the decentralized local authorities; mayors and local councilors are their elected representatives, whose legitimacy is democratic.

These two forms of authority differ in resources, incentives and temporal horizons. Conflating them in territorial health governance, as often happens in One Health arrangements, overlooks a fundamental political tension [22]. Accordingly, “local authorities” refers exclusively to municipalities and departments, and “deconcentrated services” to the state's technical services at regional and departmental levels.

### 2.3 Formal mandates and de facto roles

To separate what local authorities are formally mandated to do from what they effectively do, we draw on Bossert's notion of decision space [23], understood here as the sphere of action that actors effectively exercise irrespective of the formal mandates assigned to them. We distinguish (i) formal mandates explicitly assigned by law, in a health sector or in an adjacent domain, and (ii) de facto One Health roles: functions exercised under non-health mandates, or informally without any mandate, that influence the determinants of health risks but are not recognized as such in One Health frameworks.

## 3 Methods

### 3.1 Study design and data sources

We conducted a single-case qualitative institutional analysis in Senegal, with four sector-level units of analysis: human, animal, environmental and plant health. We drew on two sources of data. The first was a documentary analysis of legal and regulatory texts (listed in Table 1). Documents were selected if they were in force at the time of analysis and defined sectoral organization, the mandates of local authorities, or the implementation of One Health. The second was the authors' field experience: all are technical experts who have been members of the One Health Platform for three to five years. This experience guided the selection and interpretation of the documents, and the authors' field notes supported the analysis. No interviews were conducted.

**Table 1:** Legal and regulatory text sources analyzed.

**Document**	**Sector(s) / topic**	**Use in the analysis**
Law n°2013–10 of 28 December 2013, General Code of Local Authorities (Act III of decentralization)	All sectors; local authorities	Formal mandates of municipalities and departments
Law n°2023–15 of 2 August 2023, Environmental Code	Environmental health	Mandates in waste, illegal dumping, impact assessments
Law n°2018–25 of 12 November 2018, Forestry Code	Environmental health	Distribution of forest management responsibilities and revenues
Decree n°2024–845 on the National Health Security Council (HCNSS) One Health	One Health governance	Architecture of national and regional One Health bodies
Decree n°2024–959 on the attributions of the Minister of Urban Planning, Local Authorities and Land Management	Local authorities; urban planning	Ministerial responsibilities for local authorities
Deconcentration Charter, Official Journal n°7361 of 25 September 2020	Deconcentration	Contractual arrangements between State and local authorities

### 3.2 Analytical approach

For each sector, we extracted from the sources: the organization of the deconcentrated state services; the explicit mandates of local authorities; the related mandates and de facto roles through which local authorities influence determinants of health risks (e.g., waste, markets, slaughterhouses, stray animals, pastoral resources, urban planning). The sector-level results were then compared across the four sectors to identify common patterns, and interpreted using the Type I/Type II distinction of multi-level governance [19]. Table 2 summarizes the analytical grid, with its dimensions, the questions asked of each source and the coding categories.

**Table 2:** Analytical grid.

**Dimension**	**Question asked of each source**	**Coding categories**
Deconcentrated architecture	Who holds technical authority in the sector, and through which territorial levels?	Regional / departmental services; hierarchical oversight
Formal mandates of local authorities	Does the law assign a mandate to municipalities or departments in this sector?	Explicit / related (adjacent domain) / absent
Related mandates and de facto roles	Which local functions influence determinants of health risks (waste, markets, slaughterhouses, stray animals, pastoral resources, urban planning, community mobilization)?	Formal in an adjacent domain / informal; evidence base (document, literature, experiential)

## 4 Results: local authorities across the four sectors

### 4.1 Human health: decentralized responsibilities under technical oversight

Senegal's health system has been progressively decentralized through Acts I, II, and III. This dynamic has shaped both the organization of healthcare delivery and the management of pharmaceuticals, through an articulation between local responsibilities and national regulatory mechanisms. These legal frameworks assign explicit mandates to municipalities and departments in human health: (i) the construction, equipment, and maintenance of health posts and health centers; (ii) support for the management of primary healthcare facilities; and (iii) participation in the financing of Universal Health Coverage through local subsidies and community-based solidarity mechanisms [24].

However, the management and distribution of essential medicines are not directly entrusted to local authorities [25]. Their role is limited to the provision of local logistical support and community mobilization.

This configuration reflects the limited autonomy of local authorities, which operate under the technical oversight of deconcentrated health services. It amounts to a partial and functionally constrained decentralization: local authorities assume operational responsibilities without equivalent strategic decision-making power.

### 4.2 Animal health: a deconcentrated sector with indirect local levers

Veterinary public health is coordinated by the Department of Veterinary Services, which relies on a network of deconcentrated state services structured across three territorial levels [26]. This is a territorialization grounded in deconcentration, whereby the central administration extends hierarchically across the national territory.

Act III of decentralization does not formally transfer animal health mandates to local authorities. Their mandates nevertheless cover several related domains that directly influence livestock systems and, consequently, animal health [24], [27]: (i) pastoral and land resources (grazing areas, transhumance corridors, water points) fall under the responsibility of municipal and departmental councils; (ii) municipal slaughterhouses are legally under municipal jurisdiction, whereas sanitary inspection remains the responsibility of deconcentrated veterinary services. This creates a grey area of shared governance and constitutes the most direct interface between mayoral authority and veterinary public health; (iii) markets, including livestock markets, are managed and maintained by municipalities, which contributes to food safety [28] and (iv) the control of stray animals, particularly in the prevention of priority zoonoses such as rabies, constitutes a practical entry point for territorial involvement in One Health governance, even in the absence of explicit formal mandates.

This configuration generates an institutional blind spot, which is particularly problematic for the management of zoonotic diseases, as these require fine-grained coordination at the subnational level.

### 4.3 Environmental health (wildlife, water and forestry, urban planning): partial mandates, limited integration

Environmental health is an essential component of the One Health approach, encompassing the interactions between ecosystems and human and animal health [29]. In Senegal, this sector is organized according to a deconcentration logic that is even more pronounced than in the animal health sector, leaving limited scope for the formal involvement of local authorities.

Environmental health is primarily managed by two central institutions responsible for nature conservation and for the management of protected areas and classified forests. Their deconcentrated entities may involve local authorities in consultations on the environmental impact assessments of projects implemented within their jurisdictions [29]. The current Forestry Code distributes responsibilities in environmental management and natural resource governance between departments and municipalities, in line with Act III, and provides for a more equitable sharing of forest revenues between the State and local authorities [30]. This is a notable advance, but it concerns the management of forest resources rather than the surveillance of health risks associated with wildlife, an interface that is nonetheless critical from a One Health perspective.

Municipalities also hold explicit responsibilities in urban sanitation and public hygiene, in coordination with the ministries in charge of Health and Environment [12], and the Environmental Code assigns them household waste management and the monitoring of illegal dumping [29]. These mandates offer some of the most direct entry points for mayoral involvement in preventing environmental health risks, particularly through waste management and market regulation, yet they remain insufficiently integrated with national One Health frameworks and are too narrow to address the broader environmental health challenges underpinning the One Health approach.

### 4.4 Plant health: largely outside local authorities' scope

Plant health, covering the protection of plants against pests and diseases that may affect food security and biodiversity [31], is a frequently overlooked interface within the One Health approach. Pesticide use exposes farming and neighboring populations to toxicological risks, while crop losses caused by pests have significant implications for food and nutritional security. The Department of Plant Protection is responsible for phytosanitary control and preventing the introduction of harmful organisms. It operates a national agricultural surveillance and early warning system and organizes collective pest control campaigns, relying on producer organizations and village-level control committees [31]. This system is supported by a network of regional and departmental services that implement the mandates of the central administration at their respective levels, as well as by phytosanitary control posts at land borders, airports, and ports.

Local authorities are largely absent from formal plant health governance arrangements. No specific phytosanitary competencies have been transferred to them [24]. Their involvement is limited to logistical support during treatment campaigns and to public awareness-raising activities, remaining informal functions that confer no decision-making authority.

### 4.5 Cross-sectoral synthesis

Table 3 summarizes, for each sector, the deconcentrated architecture, the formal mandates of local authorities and their de facto roles. A common pattern emerges. In all four sectors, technical authority lies with vertically organized deconcentrated services. Local authorities hold explicit mandates in human health and in sanitation and waste management, but none in animal or plant health. The critical interfaces for One Health governance (wildlife surveillance as a reservoir of zoonotic diseases, pesticide management, monitoring of degraded ecosystems, zoonoses control) are organized outside their scope. This sectoral fragmentation limits the capacity to capture the ecological determinants of health risks within an integrated framework.

**Table 3:** Formal mandates and de facto roles of local authorities by sector.

Sector	Deconcentrated state architecture	Formal mandates of local authorities	Related mandates and de facto roles influencing health risk determinants	Overall position
Human health	Deconcentrated health services exercising technical oversight	Construction, equipment and maintenance of health facilities; support to primary care management; participation in UHC financing	Logistical support and community mobilization for medicines; no role in their management or distribution	Formal but functionally constrained
Animal health	Department of Veterinary Services and deconcentrated services across three territorial levels	None specific to animal health (no transfer under Act III)	Pastoral and land resources; municipal slaughterhouses (inspection by veterinary services); market and livestock market management; waste management; stray animal control	Formally absent; influential de facto
Environmental health	Central institutions for nature conservation, protected areas and forests, with deconcentrated entities	Household waste management and illegal dumping; urban sanitation and public hygiene; shared forest management and revenue; consultation on impact assessments	Urban planning and spatial regulation (zoning of livestock areas, buffer zones); wildlife health surveillance remains outside their scope	Partial; weakly integrated in One Health
Plant health	Department of Plant Protection; regional and departmental services; border control posts	None (no phytosanitary competencies transferred)	Logistical support in treatment campaigns; public awareness-raising (informal)	Largely absent; informal support only

## 5 Discussion

### 5.1 Institutional design, not capacity, explains the marginalization of local authorities

Regardless of the health dimension considered, Senegalese local authorities remain institutionally peripheral to One Health governance arrangements. They hold territorial legitimacy and formal competencies in primary healthcare, but their positioning with regard to zoonotic and other risks involving animal and environmental dimensions is not defined in existing regulatory frameworks. This marginalization does not appear to result from a deliberate policy choice. It stems from a governance architecture built on two parallel logics that have never been formally articulated: a deconcentrated state logic organized through vertical sectoral silos, and a decentralized logic structured around horizontally defined territorial mandates. The silos are replicated at each territorial level, with only ad hoc horizontal coordination under administrative authorities (governors and prefects), whereas elected local officials are, by institutional design, meant to aggregate these sectors within a territory in service of a shared population. It is precisely in this space of missed articulation that the analytical contribution of this article lies.

The limited involvement of local authorities could be interpreted as a capacity issue, a technical or financial deficit that training and additional resources would address. This reading is not without merit, but it is insufficient. One Health arrangements in Senegal have been developed according to a Type II multi-level governance logic (functional, intersectoral, and nationally driven) without being anchored to Type I territorial jurisdictions such as municipalities and departments [19]. Local authorities have not been inadvertently overlooked; they are simply not conceived as relevant actors within this governance model.

The Senegalese case is distinctive because the country has a relatively advanced decentralized architecture within the West African context [15], making this institutional gap all the more salient.

### 5.2 An unrecognized One Health function: from formal mandates to de facto roles

Local authorities already perform, in their day-to-day management, functions that objectively fall within the scope of the One Health approach, without the corresponding label, resources or integration into formal governance arrangements. Urban solid waste management, a formal municipal responsibility [24], is directly linked to the prevention of rabies, the leading priority zoonosis in Senegal: degraded environments with accessible waste promote the concentration of stray dogs, the primary source of transmission of this disease [32].

The management and maintenance of markets, particularly livestock markets and points of sale for animal products, contribute to food safety, a core dimension of the One Health approach [28]. Urban planning and spatial regulation (zoning of livestock areas, establishment of buffer zones between human settlements and pastoral areas) also constitute preventive intervention levers at the human–animal–environment interface, which mayors exercise without their public health relevance being formally recognized.

This informal and unrecognized One Health governance corresponds to the de facto decision space described by Bossert: a sphere of action that actors effectively exercise independently of the formal mandates assigned to them [23]. It is an untapped resource and a foundation on which more formalized integration could be built. Separating formal mandates from de facto roles (Table 3) also shows that the territorial level, namely municipalities and departments, is a scale at which the sectoral fragmentation inherent to One Health could be overcome in a legitimate and sustainable manner.

### 5.3 Conditions for integrating local authorities

Senegal has simultaneously deepened its decentralization process and strengthened its One Health institutional architecture without explicitly articulating these two dynamics [7], [24]. The Deconcentration Charter nevertheless provides for contractual arrangements between the State and local authorities to coordinate their actions and pool resources [33]. Because health risks emerge within territories administered by elected officials who lack both explicit mandates and sufficient resources to respond, a governance gap results. Three key conditions emerge to bridge this gap and support the sustainability of territorial One Health governance:

- **Formal recognition**: Regulatory frameworks should be revised to recognize explicitly the role of local authorities in preventing and managing health risks at the human–animal–environment interface. Without a legal foundation, their involvement depends on the individual commitment of elected officials and remains vulnerable to local political change.
- **Transfer of dedicated resources**: Responsibilities without resources remain largely symbolic [22]. Integration of local authorities into One Health governance requires mechanisms to mobilize dedicated human, financial, and logistical capacities. The experience of community-based health insurance schemes, whose expansion has been supported through state financial assistance to municipalities, provides a relevant and transferable precedent.
- **Functional inter-level coordination mechanisms**: Bridging the national One Health logic with territorial governance requires clearly defined institutional interfaces. The *CRD and CDD* could play this role, provided they evolve toward a more operational and preventive posture, systematically integrating local elected officials as decision-makers rather than mere participants. The *CRSS-OH*, if effectively operationalized with co‑leadership between elected officials and governors, could be the appropriate regional level for such coordination and strengthen the democratic legitimacy and territorial anchoring of these mechanisms.

Building on these existing roles, local authorities could contribute to One Health in concrete ways: relaying unusual animal mortality or suspect cases observed at markets and slaughterhouses to veterinary and health services (early detection of zoonotic risks); designating community focal points to report health signals (community-based surveillance); improving waste collection, sanitation and hygiene services and controlling stray animals to support rabies prevention; maintaining water, drainage and slaughterhouse infrastructure and enforcing hygiene rules in livestock markets; integrating health risks into local development and contingency plans (local preparedness and response); and bringing local risk information to regional One Health committees. These are proposals derived from our analysis; technical functions such as sanitary inspection and vaccination would remain with deconcentrated services.

The ongoing development of Senegal's second National Action Plan for Health Security offers a concrete policy window to advance this reform and may provide lessons for other West African countries with comparable decentralized architectures.

### 5.4 Limitations and perspectives

This study is based on a documentary analysis of legal, policy and scientific sources. The de facto roles identified derive from local authorities' mandates in adjacent domains and from the literature, and on the authors' field experience, which was not collected through a systematic protocol; they should be validated through field studies, such as interviews with mayors, local councilors and deconcentrated services, or case studies of selected municipalities.

The heterogeneity of municipalities, in terms of resources, agro-ecological profiles, administrative capacities, and local political dynamics, implies that the conditions for territorial integration of the One Health approach vary significantly across contexts. A single model of territorial One Health governance would therefore be limited; a differentiated approach appears more realistic.

Local political will cannot be overlooked. Integrating local authorities into One Health governance requires elected officials to take ownership of these issues, which is not guaranteed in a context where animal and environmental health are rarely perceived as electoral priorities. Communication and awareness-raising strategies targeting local elected officials could be a relevant entry point.

Finally, the articulation between Senegalese territorial governance and West African regional dynamics deserves particular attention. Transhumance corridors, transboundary epizootics, and trade flows of animal products extend beyond both municipal and national boundaries. Territorial One Health governance in Senegal will need to align with regional mechanisms led by the Economic Community of West African States (ECOWAS); otherwise, it will remain incomplete in addressing health risks that, by nature, transcend administrative boundaries.

## 6 Conclusion

This article shows that the limited involvement of local authorities in One Health governance in Senegal stems less from capacity deficits than from a structural misalignment between a deconcentrated, sectoral, and vertical state logic and a decentralized, territorial, and horizontal system of governance. While One Health governance is expanding toward the local level, local authorities remain at its institutional periphery, even though their mandates in waste management, public hygiene, urban planning, markets and natural resources already shape health risk determinants. Formal recognition, dedicated resources and functional inter-level coordination would allow this underutilized potential to be converted into more integrated and territorially anchored One Health governance, a pillar that remains insufficiently developed within the One Health approach.

## CRediT authorship contribution statement

**Mouhamadou Wilane:** Writing – review & editing, Writing – original draft, Conceptualization. **Khadija Sandembou Diop:** Writing – original draft. **Gérome Sambou:** Writing – original draft.

## Declaration of competing interest

The authors declare that they have no known competing financial interests or personal relationships that could have appeared to influence the work reported in this paper.

## Data Availability

The authors are unable or have chosen not to specify which data has been used.
